# Determining the optimal pulse number for theta burst induced change in cortical excitability

**DOI:** 10.1038/s41598-021-87916-2

**Published:** 2021-04-22

**Authors:** Daniel M. McCalley, Daniel H. Lench, Jade D. Doolittle, Julia P. Imperatore, Michaela Hoffman, Colleen A. Hanlon

**Affiliations:** 1 Department of Psychiatry and Behavioral Sciences, Medical University of South Carolina, Charleston, SC USA; 2 Department of Neurosciences, Medical University of South Carolina, Charleston, SC USA; 3 Department of Neurology, Medical University of South Carolina, Charleston, SC USA; 4 Departments of Cancer Biology, Physiology and Pharmacology, Wake Forest School of Medicine, Winston-Salem, NC 27106 USA

**Keywords:** Neuroscience, Motor cortex

## Abstract

Theta-burst stimulation (TBS) is a form of non-invasive neuromodulation which is delivered in an intermittent (iTBS) or continuous (cTBS) manner. Although 600 pulses is the most common dose, the goal of these experiments was to evaluate the effect of higher per-dose pulse numbers on cortical excitability. Sixty individuals were recruited for 2 experiments. In Experiment 1, participants received 600, 1200, 1800, or sham (600) iTBS (4 visits, counterbalanced, left motor cortex, 80% active threshold). In Experiment 2, participants received 600, 1200, 1800, 3600, or sham (600) cTBS (5 visits, counterbalanced). Motor evoked potentials (MEP) were measured in 10-min increments for 60 min. For iTBS, there was a significant interaction between dose and time (F = 3.8296, *p* = 0.01), driven by iTBS (1200) which decreased excitability for up to 50 min (t = 3.1267, *p* = 0.001). For cTBS, there was no overall interaction between dose and time (F = 1.1513, *p* = 0.33). Relative to sham, cTBS (3600) increased excitability for up to 60 min (t = 2.0880, *p* = 0.04). There were no other significant effects of dose relative to sham in either experiment. Secondary analyses revealed high within and between subject variability. These results suggest that iTBS (1200) and cTBS (3600) are, respectively, the most effective doses for decreasing and increasing cortical excitability.

## Introduction

Human Theta Burst Stimulation (TBS) is a form of transcranial magnetic stimulation (TMS) in which TMS pulses are delivered in triplets that occur 5 times per second. Initial support for TBS arose from in vitro electrophysiology research which demonstrated that theta burst stimulation to the hippocampus can induce long-term potentiation (LTP)^1–4^. In 2005, Huang and colleagues delivered theta burst stimulation to humans via a conventional figure of eight TMS coil^5^. Since then TBS has been widely embraced by researchers and clinicians due to its relatively high efficiency as a brain stimulation tool^6^. It also received Food and Drug Administration (FDA) clearance for use in treating medication resistant major depressive disorder (MDD) in 2018. Amidst this enthusiasm however, several questions remain unanswered regarding the optimal parameters of TBS.

One open question involves the optimal number of pulses that should be applied to the cortex during each session. The majority of studies apply 600 pulses of TBS to the cortex in either a continuous (cTBS) or intermittent (iTBS) manner. The initial study in 9 individuals demonstrated that 600 pulses of iTBS amplified cortical excitability and 600 pulses of cTBS attenuated excitability^5^. A subsequent study in 2010 by Gamboa et al. demonstrated that doubling the number of TBS pulses led to a paradoxical reversal of these effects—1200 pulses of iTBS decreased excitability and 1200 pulses of cTBS increased excitability^7^. These results, however, have been inconsistent^8^. In addition to 600 and 1200 pulses, some groups have chosen 1800 pulses^9,10^ and 3600 pulses^11^ as a TBS dose.

To date, however, there has not been a prospective, randomized, sham-controlled evaluation of the these emerging TBS protocols and their impact on cortical excitability. The primary goal of this study was to evaluate the effect of escalating pulse number on TBS-associated modulation of the motor cortex. As with most of the studies in this field, the motor cortex was chosen as the model system to modulate cortical excitability, wherein the amplitude of motor evoked potentials (MEPs) was the primary dependent measure. Healthy, right-handed adults were recruited from the community without regard to their genotype in order to maximize generalizability to a heterogenous population of young to middle aged adults.

## Methods

### Overview

Sixty right-handed individuals from 21 to 35 years old with no history of neurologic injury were recruited from the Charleston, SC metropolitan community to participate in one of two non-invasive brain stimulation experiments. These experiments were approved by the Medical University of South Carolina Institutional Review Board and were performed in accordance with Declaration of Helsinki on Ethical Principles for Medical Research. Each participant gave informed written consent prior to study participation.

In Experiment 1, at each visit, participants received one of three doses of intermittent TBS (600, 1200, 1800 pulses) or sham TBS (600 pulses) in a randomized order (n = 30, 20F, 10 M, 24.4 ± 3.7, mean age ± SD)**.** In Experiment 2, at each visit, participants received one of four doses of continuous TBS (600, 1200, 1800, and 3600 pulses) or sham TBS (600) in a randomized order (n = 30, 18F, 12 M, 25.0 ± 3.4, mean age ± SD). Each visit was separated by at least 2 days to prevent carry-over effects.

Electromyography (EMG) was used at each visit to record MEPs of the abductor pollicis brevis (APB) muscle in the right hand before and after a dose of TBS. Handedness was confirmed using the Edinburgh Handedness Inventory (86.7 ± 14.1; mean EHI score ± SD)^12^.

At each study visit, Ag–AgCl surface electrodes (Natus Neurology Incorporated, Middleton, Wisconsin) were placed with a belly-tendon montage on the APB muscle of the right hand. The TMS coil was then positioned in a posterior-anterior (PA) direction at a 45-degree angle over the left motor cortex. To determine the motor ‘hotspot’ (defined here as the cortical location evoking the largest EMG response in the target muscle), a series of single pulses of TMS were applied with a grid-based searching system starting from the C3 location (EEG 10–20 system) and extending in 1–2 cm increments in 4 directions with a minimum 4 s interval between TMS pulses. All TMS procedures were performed using Magventure’s X100-Magoption equipped with a COOL-B65 figure-of-eight coil with an outer winding diameter of 75 mm (Magventure Inc., Farum, Denmark). Raw EMG signals from a CED four-channel electrode adapter box were amplified, band-pass filtered (100–10,000 Hz) and sampled (5000 Hz) using a CED 1902 amplifier, a CED Micro1402 analog-to-digital converter, and CED Spike 2 software (Cambridge Electronics Design, United Kingdom). The motor hotspot for each participant was recorded using neuronavigation software (Brainsight; Rogue Research Incorporated, Montreal, Quebec, Canada) such that the same cortical location could be accurately stimulated across timepoints and study visits.

**Figure 1:**
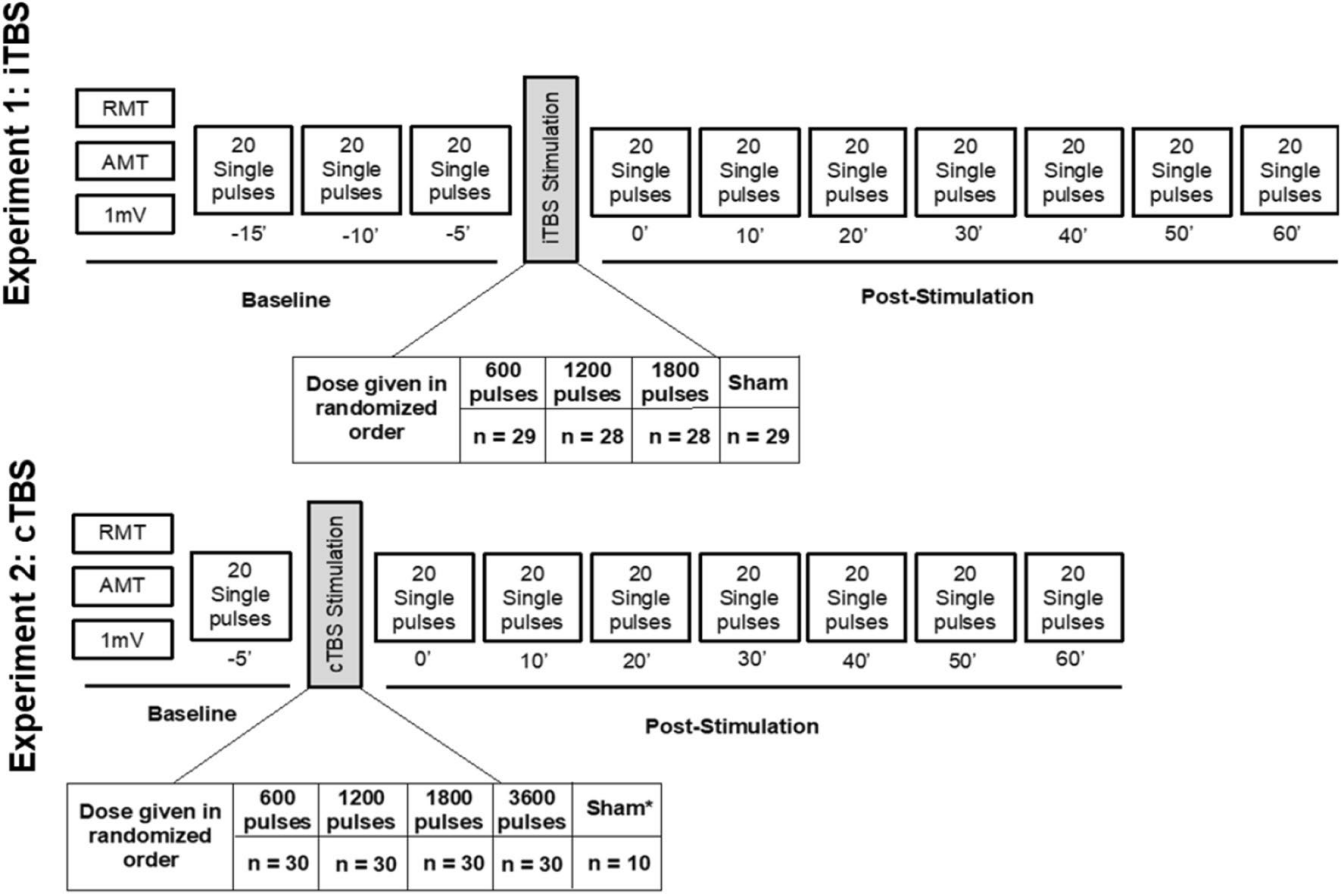
Diagram of experimental design for iTBS and cTBS experiments. This diagram represents the flow of assessments performed on a given visit. Each visit began with resting, active and 1 mV thresholds. Next, 20 baseline MEPs were collected prior to TBS administration (for participants in the iTBS experiment this was performed 3 times). A dose of iTBS or cTBS was administered at 80% aMT. Directly following stimulation 20 MEPs were collected and repeated at 10-min intervals thereafter. Only a subset of participants during Experiment 2 received sham stimulation.

After the localization of this motor ‘hotspot’, a series of standard metrics were acquired including the resting motor threshold (rMT), active motor threshold (aMT), and the stimulator intensity needed to reliably obtain an average peak-to-peak amplitude of approximately 1 mV (SI_1mV_) (Fig. 1). All three measures were found using parameter estimation by sequential training (PEST), an automated algorithm used to determine TMS thresholds^13^. During the rMT and 1 mV threshold, participants were instructed to relax their right hand on a pillow. During the aMT, participants were instructed to touch their thumb to their pointer finger (making an O.K. sign), thereby slightly flexing the APB muscle. To standardize measurement thresholds, an automated TMS-EMG-PEST feedback system was programmed using Spike2 software (Cambridge Electronic Design Limited, Cambridge, England). Baseline cortical excitability was determined by applying 3 blocks of 20 single pulses of TMS separated by 5 minute intervals (Experiment 1) or 1 block of 20 single pulses of TMS (Experiment 2) at the predetermined 1 mV threshold. An additional 5-min rest interval was introduced between the baseline measurement and application of TBS.

Following TBS administration (see details below), blocks of 20 MEPs were collected at 0, 10, 20, 30, 40, 50, and 60 mins (Fig. 1). During periods of stimulation and recording, participants were instructed to keep their hand still and relaxed. To maintain a relatively fixed level of vigilance though the 60 min session, in between the EMG sampling intervals, participant viewed episodes from a nature documentary (Planet Earth, Public Broadcasting System, 2006).

### Theta-burst stimulation protocols

#### Experiment 1—intermittent TBS (iTBS)

iTBS was administered at 80% of each participant’s aMT in a burst-firing pattern (3 pulses at 50 Hz) for a 2 s train, followed by an 8 s period of rest. During each visit, participants received one of three intermittent theta-burst protocols or a sham stimulation protocol in a randomized order: (1) 600 pulses (190 s), (2) 1200 pulses (380 s), (3) 1800 pulses (570 s), and (4) a sham stimulation protocol^14^ in which a 3 cm foam spacer was placed in between the coil and the participant’s scalp, thereby increasing the coil-to-cortex distance while preserving the sensory aspects of iTBS.

#### Experiment 2—continuous TBS (cTBS)

cTBS was administered at 80% of each participant’s aMT in a burst-firing pattern (3 pulses at 50 Hz) with a repeated frequency of 5 Hz (200 ms intervals). During each visit, participants received one of four continuous theta-burst protocols in a randomized order: (1) 600 pulses (40 s), (2) 1200 pulses (80 s), (3) 1800 pulses (120 s), (4) 3600 pulses (1800 pulses, 60 s break, 1800 pulses). (5) A sham stimulation protocol^15^ which included electrodes placed at the hairline (Natus Neurology Incorporated, Middleton, Wisconsin). Sham data were collected from 10 of the individuals.

### Data analysis

#### TMS parameter stability

Test–retest reliability of the TMS parameters was evaluated by assessing the degree of absolute agreement as measured by across session intraclass correlation coefficient (2-way mixed-model, alpha = 0.05) for the following measurements: rMT, aMT, SI_1mV_ threshold, and average baseline MEP amplitude (SPSS v.23, IBM). Intraclass correlation coefficients (ICCs) were interpreted as follows: excellent reliability ≥ 0.75; moderate to good reliability 0.74–0.40; and poor reliability < 0.40^16^.

#### Effect of TBS dose on change in motor cortex excitability

All MEP amplitudes were quantified using MAVIN, an automated open source tool for EMG quantification^17^. Using the TMS trigger artefact, MAVIN identifies a window of EMG activity directly following the test pulse and calculates the peak-to-peak amplitude of each MEP. Any visual artefacts were rejected before data were exported from MAVIN to excel for data organization. MEPs that did not elicit a response greater than 0.3 mV outright were removed from analysis. Following removal of low-amplitude MEPs, MEPs which reflected a change from baseline of ± 2.5 STDEV from the mean of all post-TBS observations were excluded^18^. For quantification of excluded MEPs across treatments and subjects, see Supplementary Tables. S1-2, respectively. For frequency distribution of MEP amplitude pre and post TBS, the reader is further referred to Supplementary Fig. S1.

**Figure 2:**
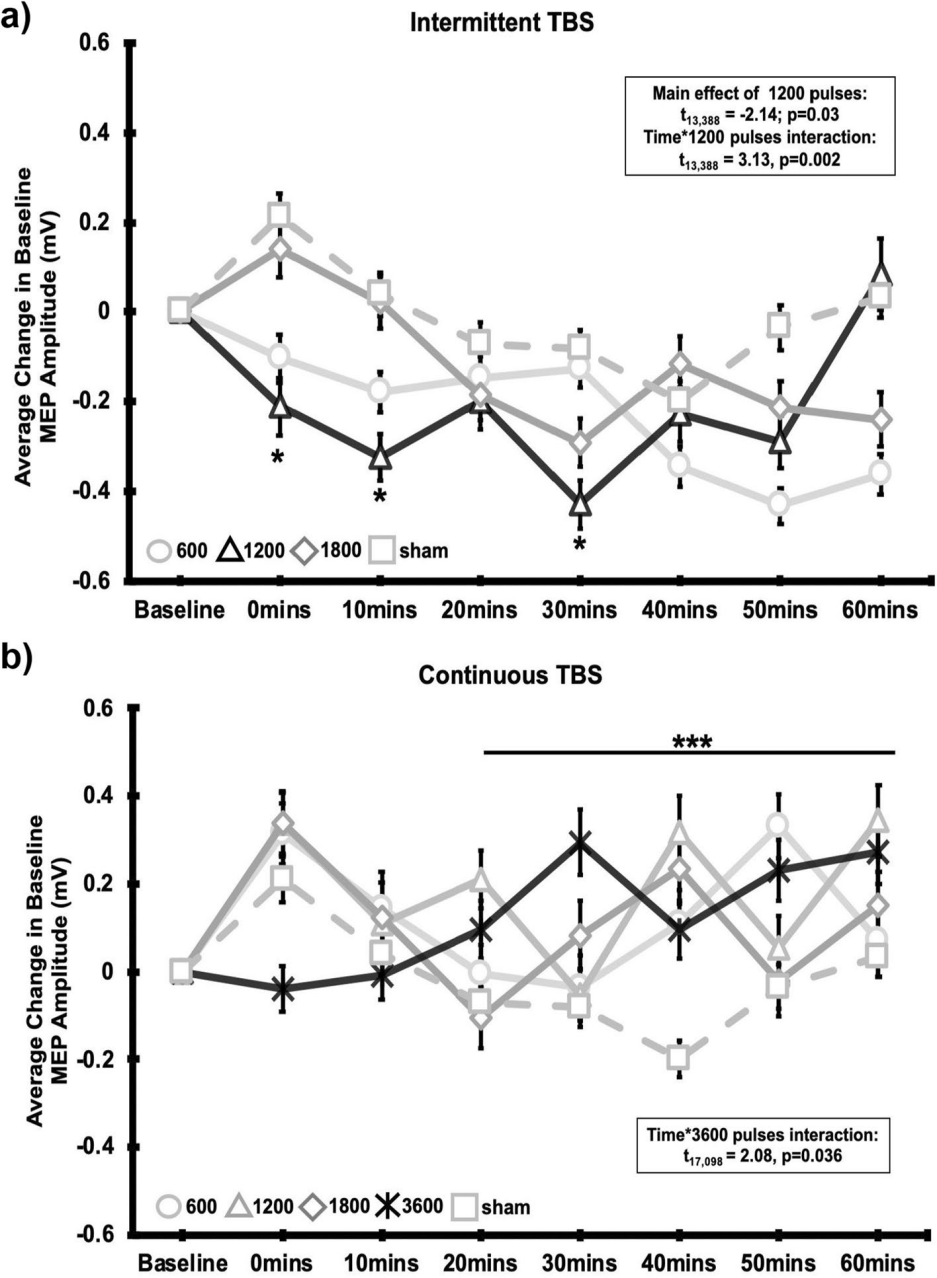
Change in MEP amplitude over time. Line graph showing the (**a**) Effect of iTBS (Experiment 1) doses and sham on average change from baseline MEP over time and (**b**) Effect of cTBS (Experiment 2) doses and sham on average change from baseline MEP over time. Error bars represent ± SEM (standard error of the mean). Average change in MEP for post iTBS and cTBS time points were calculated by subtracting the average baseline MEP amplitude from all MEPs post-treatment. Shapes represent each MEP assessment time point following the 600 pulses (circle), 1200 pulses (triangle), 1800 pulses (diamond), and 3600 pulses (X’s) of iTBS and cTBS. TBS protocols that were significantly different than sham stimulation over time are depicted by black lines. Sham stimulation is represented with a dotted line and squares for each time point. The results of the LME are embedded in the graph above.

A linear mixed-effects model was created for each experiment using the MEP data, wherein MEP is nested within timepoint (Baseline, 0, 10, 20, 30, 40, 50, and 60 min) and timepoint is nested within dose (iTBS: 600, 1200, 1800 pulses or sham; cTBS: 600, 1200, 1800, 3600 or sham) for each participant (Supplementary Fig. S2). The results are shown in Fig. 2 (described below), to be comprehensive, however, we’ve also included a supplemental figure which shows these data with no outlier MEPs excluded (Supplementary Fig. S4).

These models were estimated using the Matlab function ‘fitlme’ with REML estimation predicting change from baseline MEP amplitude following each TBS dose with respect to sham (Matlab, R2020a, The MathWorks Inc). Each model fit fixed-effects for TBS (iTBS: sham, 600, 1200, 1800 doses and cTBS: sham, 600, 1200, 1800, 3600) and time (minute, as a continuous variable ranging from 0 to 60) as well as their interaction, and covaried each individual’s rMT. Treatment included the sham condition for both iTBS and cTBS. As described above, the following random effects were included: the mean change from baseline was allowed to vary by subject, treatment within subject, time within treatment, and individual MEP within time independently. Given that most experiments in the TBS field investigate a single dose of TBS, univariate analyses for each dose relative to time were performed within the multivariate linear mixed model.

#### Exploratory analysis of variability

Given prior reports of individual variability in TBS studies, we performed a qualitative secondary analysis to assess individual variability in TBS response patterns. We classified each individual’s response to TBS as ‘excitatory’, ‘inhibitory’, or ‘no change’. In line with Perellon-Alfonso et al.^19^, this was done by calculating the sum of all MEPs in the 60 min after TBS and dividing the by the total number of MEPs collected. An ‘excitatory’ response was defined as greater than 20% increase in MEP amplitude, an ‘inhibitory’ was defined as a greater than 20% decrease in MEP amplitude. Otherwise, the response was designated ‘no change’.

## Results

### Baseline measurements

There was high test–retest reliability of the baseline measurements of rMT (Exp 1: ICC = 0.964, 95% CI = 0.935–0.982; Exp 2: ICC = 0.965, 95% CI = 0.940–0.982), aMT (Exp 1: ICC = 0.958, 95% CI = 0.925–0.979; Exp 2: ICC = 0.935, 95% CI = 0.887–0.966), SI_1mV_ (Exp 1: ICC = 0.975, 95% CI = 0.954–0.987; Exp 2: ICC = 0.965, 95% CI = 0.938–0.982) and average baseline MEP amplitude (Exp 1: ICC = 0.747, 95% CI = 0.549–0.873; Exp 2: ICC = 0.599, 95% CI = 0.291–0.794) as revealed by intraclass-correlation coefficient (Supplementary Figs. S5-S8). Mean motor thresholds and mean baseline MEP amplitudes are shown in Table 1. Active motor thresholds were 10.3% less than rMT for iTBS and 10.1% less than rMT for cTBS. 1 mV thresholds were 15.3% greater than rMT for iTBS and 16.3% greater than rMT for cTBS.

**Table 1:** Motor thresholds and baseline MEP amplitudes. Mean (± SD) resting motor threshold (%MSO), active motor threshold (%MSO), 1 mV stimulus intensity threshold (% MSO), and recorded baseline MEP amplitude (mV).

		V1	V2	V3	V4	V5
**Experiment 1: iTBS**	Resting Motor Threshold	48.62 ± 9.28	50.48 ± 10.81	47.93 ± 10.58	48.70 ± 9.94	**N/A**
	Active Motor Threshold	42.28 ± 9.07	45.48 ± 9.81	43.18 ± 9.34	43.67 ± 9.25	
	1 mV Stimulus Intensity	57.38 ± 11.67	58.03 ± 14.13	55.29 ± 12.50	54.85 ± 11.47	
	Baseline MEP amplitude (1 mV)	1.57 ± 0.96	1.47 ± 0.60	1.30 ± 0.66	1.44 ± 1.05	
**Experiment 2: cTBS**	Resting Motor Threshold	51.77 ± 12.15	53.27 ± 12.20	53.32 ± 10.86	50.57 ± 10.86	48.0 ± 14.41
	Active Motor Threshold	46.10 ± 12.69	46.73 ± 11.58	48.18 ± 10.07	46.18 ± 11.06	43.27 ± 14.31
	1 mV Stimulus Intensity	60.47 ± 14.38	61.70 ± 13.98	59.61 ± 12.95	60.00 ± 14.90	55.45 ± 17.83
	Baseline MEP amplitude (1 mV)	1.54 ± 0.80	1.55 ± 0.81	1.57 ± 0.60	1.46 ± 0.59	1.52 ± 0.77

### Experiment 1—iTBS

The MEP data for each dose over time is shown in Fig. 2a. There was a significant main effect of time (F_1, 13,338_ = 5.3473, *p* = 0.02) and a significant time*dose interaction (F_3, 13,338_ = 3.8296, *p* = 0.01) relative to the sham condition. There was a main effect of 1200 pulses (t_13,388_ = − 2.1374, *p* = 0.03) as well as a time*1200 pulse interaction (t_13,338_ = 3.1267, *p* = 0.001), wherein 1200 pulses was net inhibitory relative to sham at the 0 (t_858.6_ = − 3.155, *p* = 0.002), 10 (t_1,053_ = − 4.663, *p* = 4.0 * 10^–6^), 30 (t_1,032_ = − 4.96, *p* = 8.15 * 10^–7^) and 50 (t_1,011_ = − 3.10, *p* = 0.002) minute timepoints.

There was no main effect of the 600 pulses (t_13,338_ = 0.0586, *p* = 0.95) or 1800 pulses (t_13,338_ = 1.415, *p* = 0.15), nor a time*600 pulse interaction (t_13,338_ = − 1.3564, *p* = 0.17) relative to sham. There was borderline time*1800 pulse interaction (t_13,338_ = − 1.9368, *p* = 0.053).

### Experiment 2—cTBS

The MEP data for each dose over time is shown in Fig. 2b. When all doses were considered in aggregate relative to sham, there was no main effect of time (F_1, 17,098_ = 0.0692, *p* = 0.79) or dose (F_4, 17,098_ = 0.0871, *p* = 0.97) nor a significant time*dose interaction on MEP data following cTBS (F_4, 17,098_ = 1.1513, *p* = 0.33), relative to sham.

When each does was considered independently relative to sham (as is the typical design in TBS experiments), there was a significant 3600 pulse * time interaction (t_17,098_ = 2.0880, *p* = 0.04), wherein 3600 pulses increased excitability relative to sham at the 20 (t_747.97_ = 2.465, *p* = 0.014), 30 (t_666.20_ = 4.556, *p* = 6.0 * 10^–6^), 40 (t_701.06_ = 3.908, *p* = 0.0001), 50 (t_870.28_ = 2.936, *p* = 0.003) and 60 (t_717.87_ = 3.688, *p* = 0.0002) minute timepoints.

There was no significant main effect for the other cTBS doses (cTBS (600): t_17,098_ = 0.1611, *p* = 0.87; cTBS(1200) t_17,098_ = 0.5041, *p* = 0.61; cTBS(1800) t_17,098_ = − 0.1749, *p* = 0.86; cTBS(3600): t _17,098_ = − 0.2851, *p* = 0.76), nor a significant dose*time interaction (cTBS (600) t_17,098_ = − 0.1092, *p* = 0.91; cTBS(1200) t_17,098_ = − 0.5479, *p* = 0.58; cTBS (1800) t_17,098_ = − 0.5892, *p* = 0.56).

[Note: While univariate analyses are typically only performed if there are main effects or interactions in the multivariate model, a univariate comparison of each dose relative to sham accurately mimics typical experimental design in the TBS field. While this may not be technically correct from a conservative statistical perspective, not reporting these results may do a disservice to the field.]

### Exploratory analysis of individual variability

**Figure 3:**
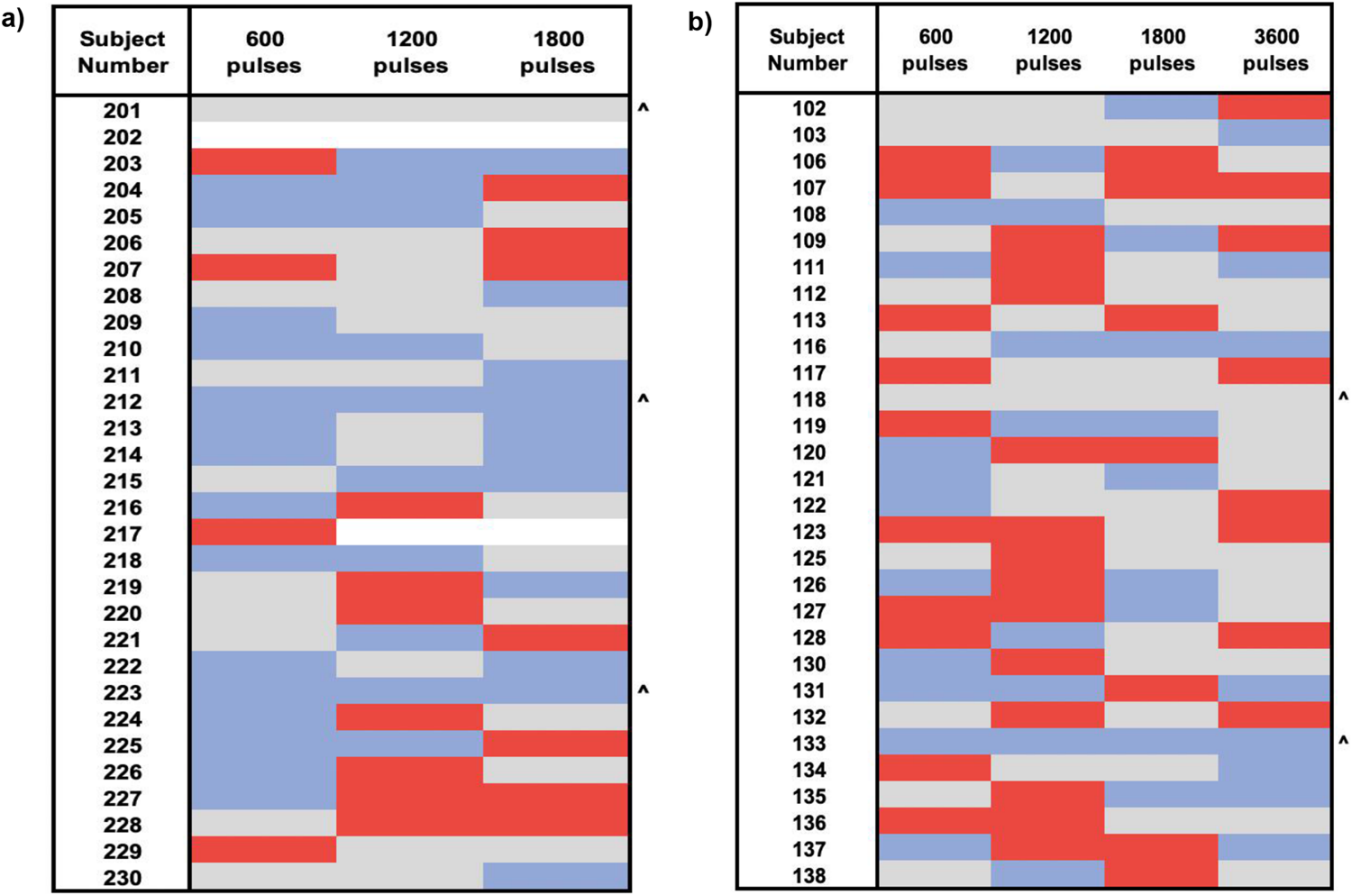
Individual Response to TBS protocols. Colored grid shows the breakdown of inhibitory (blue), excitatory (red) or no change (grey) responses following (**A**) iTBS and (**B**) cTBS doses in each individual. **^**indicates individuals who consistently responded in the same direction to all real TBS protocols.

We evaluated within-subject consistency of responding in a given manner to iTBS or cTBS. As shown in Fig. 3, only 3 individuals demonstrated a consistent response to the tested real iTBS protocols (1 consistently demonstrating no change and 2 consistently inhibitory). Similarly, only 2 individuals demonstrated a consistent response to the tested real cTBS protocols (1 consistently demonstrating no change and 1 consistently inhibitory).

## Discussion

Here we present the first parametric sham-controlled study to explore the relationship between TBS pulse number (both cTBS and iTBS) and motor cortex excitability. The goal of these experiments was to systematically evaluate the effect of TBS dose on the change in motor cortex excitability. As a refinement of previous studies, we also included an active sham condition. The primary conclusions are that (1) within iTBS protocols, 1200 pulses is significantly different from sham, resulting in a net inhibition of cortical excitability, and (2) within cTBS protocols, 3600 pulses is different than sham, resulting in a net increase in excitability. We did not, however, find significant effects of the other doses relative to sham. This may be due to a high degree of intra- and interindividual variability—a finding which has been reported by many other studies in the TBS field.

### iTBS (1200) decreases motor cortex excitability

The primary finding of our investigation is that 1200 pulses of iTBS causes a significant decrease in cortical excitability. This observation complements and extends prior work from Gamboa et al. 2010 wherein 1200 pulses of iTBS decreased cortical excitability (whereas 600 pulses had increased excitability).They also found that 1200 pulses of cTBS increased excitability (whereas 600 pulses had decreased excitability)^7^. Hsu et. al 2011 also evaluated the effect of 600 versus 1200 pulses of TBS on excitability and failed to find an effect of pulse number on excitability^8^. Notably, both of these groups used relatively small sample sizes in their analyses (*n* = 14 & 10 respectively), did not incorporate an active sham control, and one group pre-selected individuals with a Val/Val genotype for BDNF^7^. Here, with a much larger sample, we failed to find a difference between 600 pulses of iTBS or cTBS and sham, but, consistent with Gamboa and colleagues, 1200 pulses of iTBS produced a significant attenuation of cortical excitability.

### The directionality of 600 pulses of iTBS and cTBS on cortical excitability

As mentioned in the introduction, early studies presented by Huang et. al and others led to the widespread adoption of 600 pulses of TBS in manipulating motor cortex excitability. Indeed, to date, at least 139 studies (iTBS: 86; cTBS: 90; both: 37) have utilized this 600-pulse dose in their investigations of the impact of TBS on motor cortex excitability in healthy controls. On the whole, the majority of published work demonstrates an “LTP-like” effect of 600 pulses of iTBS and an “LTD-like” effect of 600 pulses of cTBS (as measured by an increase or decrease in MEP amplitude post treatment, respectively).

The current study suggests that in healthy controls, 600 pulses of cTBS or iTBS does not produce a significant change in motor cortex excitability relative to sham stimulation. In fact, iTBS tends to have a net inhibitory effect on cortical excitability—which is driven primarily by the 1200 pulse condition, and cTBS tends to have a net excitatory effect on cortical excitability—driven by an effect of 3600 pulse condition. This can be seen both through the multivariate model as well as a qualitative view of the results in Fig. 2.

While the lack of facilitation with iTBS(600) and inhibition with cTBS(600) is contrary to many previously published studies, there are several key differences between our experimental protocols and the prior work in this field. Foremost, among the previously published work (*n* = 139), only 25 studies (approximately 18%) have incorporated a sham or control condition^8,19–22^. While many of them have reported significant changes in motor cortex plasticity following TBS, relative to sham, many of the sham strategies used (stimulating alternate brain regions, tilting the coil away from the head, or applying sham tDCS) do not robustly model the somatosensory experience of receiving TMS in a given location. Indeed, in a recent, sham-controlled study (incorporating a sham that accurately mimics the somatosensory aspects of TMS), Perellon-Alfonso et al.^19^ were unable to detect a significant difference in iTBS-induced (relative to sham-induced) change in motor cortex plasticity, even when 600 pulses of iTBS were applied for 5 consecutive days. Similar active, sham-controlled studies have found no change^20^ or very small decreases^18^ in motor cortex excitability following 600 pulses of cTBS.

Further, we note that several studies pre-select participants based on intrinsic biological variables such as BDNF genotype^7,22–26^, TRPV1 mutation^27^, APOE3 mutation^28^ or COMT polymorphism^29^. Given that the current study recruited from the community and did not filter for a specific genotype, our results many not be directly comparable to these works. Lastly, we note that among the existing literature, approximately 26% of existing works have incorporated sample sizes greater than 20 young, healthy controls. Among these works, many groups have been unable to find a significant reduction in MEP amplitude following 600 pulses of cTBS^28,30–37^ or a significant increase in MEP amplitude following 600 pulses of iTBS^24,37–41^.

### Inter- and intra- subject variability

A growing body of evidence has emerged suggesting a high degree of inter- and intra-individual variability exists in response to TBS protocols. Several groups have observed variable responses to TBS in that some individuals demonstrate no change in motor cortex excitability following TBS, or an “opposite” effect of TBS wherein cTBS and iTBS produce an increase or decrease in cortical excitability, respectively^19,28,37,39,40,42–44^.

Following 600 pulses of TBS, we find that the proportion of individuals experiencing the expected inhibition or excitation following cTBS or iTBS, respectively, are comparable to proportions reported by several large-scale studies when applying similar thresholds in defining an individual’s response to TBS as excitatory or inhibitory (± 1% change in MEP amplitude after TBS treatment). Hamada et al. reports only 42% of individuals displaying the expected inhibitory response to cTBS and others report 43–52% displaying the expected excitatory response to iTBS^37^. Applying the same threshold to our data, we find a comparable 40% of individuals showing an inhibitory response to 600 pulses of cTBS, and 33% showing an excitatory response to 600 pulses of iTBS. Interestingly, a recent a position paper from Huang and colleagues has speculated that the probability of producing the expected inhibitory or excitatory response to various patterns of TMS (including TBS), may be less than 50%^45^, in line with the results presented here. Taken together, the results from our experiments, and from a number of others demonstrating substantial degrees of individual variability underscore the need for a clear path forward for future research regarding TBS, motor cortex excitability, and potential best practices.

TMS-administration related variables such as sample size, sham controls, and coil orientation are known factors that may contribute to variability. We find that the substantial variability observed here is mostly congruous with TBS experiments wherein sample sizes exceeded 20 healthy controls receiving real TBS. As such, we recommend a standard of at least 20 individuals to be enrolled in these types of studies. Further, we find that many groups either do not incorporate a sham condition or apply a sham that does not accurately mimic the somatosensory effects of active TBS, potentially unblinding participants. Given that the impact of participant expectation towards real or sham treatment remain largely unknown, we recommend that active sham systems (such as those methods reported here) become the standard in field of TBS and motor cortex excitability. Lastly, as previously mentioned, Hamada et al. 2013 and others have suggested that small changes in coil orientation may recruit distinct populations of interneurons, potentially skewing results or introducing variability. Future studies may consider incorporating contemporary neuronavigational systems to rigorously standardize coil placement across visits and across timepoints within an experiment.

## Limitations

We note several limitations in our experimental design which may limit the scope of our work. Primarily, the present studies collected motor-evoked potentials only and did not perform more complex measures such as SICI or ICF and can therefore only assess changes in corticospinal excitability rather than direct neuroplastic changes in circuits intrinsic to the motor cortex. We are therefore unable to extensively speculate on the neurobiological mechanisms which may mediate our results. Additionally, the sham condition for the two experiments was slightly different, wherein 10 individuals received surface electrode stimulation on the scalp and 30 received stimulation with a 3 cm foam padding placed between the coil and the scalp. While we do not expect that these sham conditions to produce disparate results, future research should incorporate a consistent, rigorous sham for all participants given the substantial variability in response to TBS. We also note that the current studies recruited primarily young adults. While this is seemingly the standard in the field, we cannot generalize these results to middle-aged or older adults. Lastly, our interpretations of these data are limited as they apply only to the use of TBS in a single session, rather than across consecutive days, as is often the case in studies using TBS to treat psychiatric disorders.

## Conclusion

To the best of our knowledge, this is the first sham-controlled study to parametrically investigate the relationship between multiple TBS doses (with varying pulse numbers) on motor cortex excitability. Prior to this report, only two groups had directly examined the impact of pulse number on TBS-related change in motor cortex excitability in a single, uninterrupted TBS session^7,8^. The current study reflects the results of 241 individual experiments/observations, 60 participants, and 43,080 collected motor evoked potentials. The primary results from this study demonstrate that (1) 1200 pulses of iTBS has a significant inhibitory effect on motor cortex excitability (2) 3600 pulses of cTBS has a significant excitatory effect on motor cortex excitability and (3) individual response to a given cTBS or iTBS protocol (e.g. 600 pulses) is inconsistent with the same individual’s response to another cTBS or iTBS protocol (e.g. 1200 pulses). This study supports a growing body of literature suggesting that directed modulation of cortical excitability using TBS requires a highly controlled environment and may not be ideally suited for heterogenous patient populations.

### A path forward

Given the growing popularity of TBS as a novel tool to treat psychiatric and motor disorders alike (for review, see Burke et al.^46^), we suggest that more emphasis be placed on the 1200 pulse iTBS and 3600 pulse cTBS conditions as these protocols had significant effects on cortical excitability relative to sham—even in the presence of high individual variability.

## Supplementary Information

Supplementary information.

## Data Availability

Any supporting data needed or requested by the Editorial Board Members and/or referees are or will be made available.
