# Supplementary material for: Determining the optimal pulse number for theta burst induced change in cortical excitability
Source: Sci Rep. 2021 Apr 22;11:8726. doi: 10.1038/s41598-021-87916-2 (PMC8062542; doi:10.1038/s41598-021-87916-2)
Supplement: Supplementary file 1 — Supplementary information. [file 41598_2021_87916_MOESM1_ESM.docx]

**Supplementary Information**

Determining the optimal pulse number for Theta Burst induced change in cortical excitability.

Daniel M. McCalley*^1,2^, Daniel H. Lench^*3^, Jade D. Doolittle^1^, Julia P. Imperatore^1,2^, Michaela Hoffman^1^, Colleen A. Hanlon**^1,2,4^

*Supplemental Fig. S1: Distribution of Collected MEP Amplitudes*

**a)**


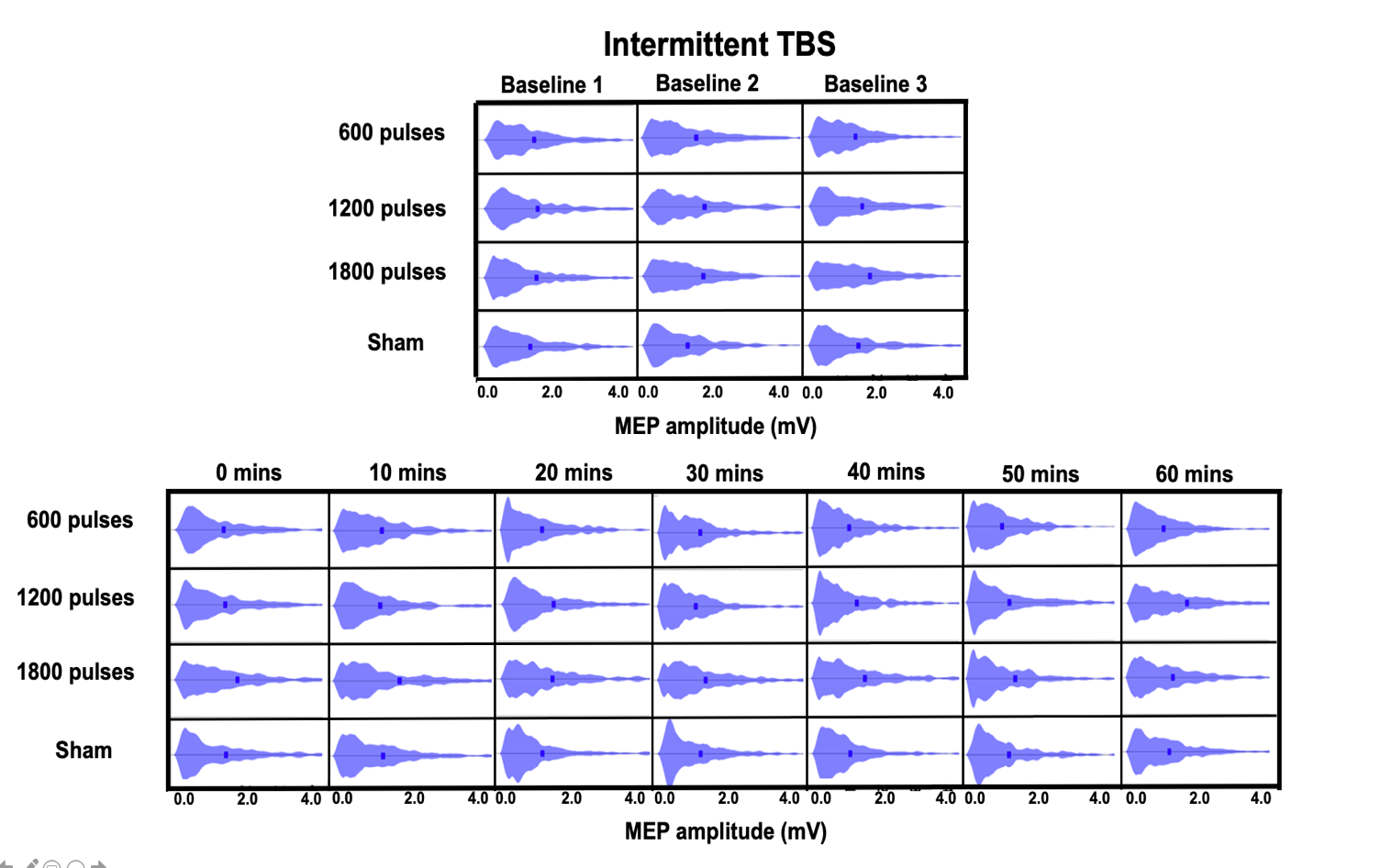


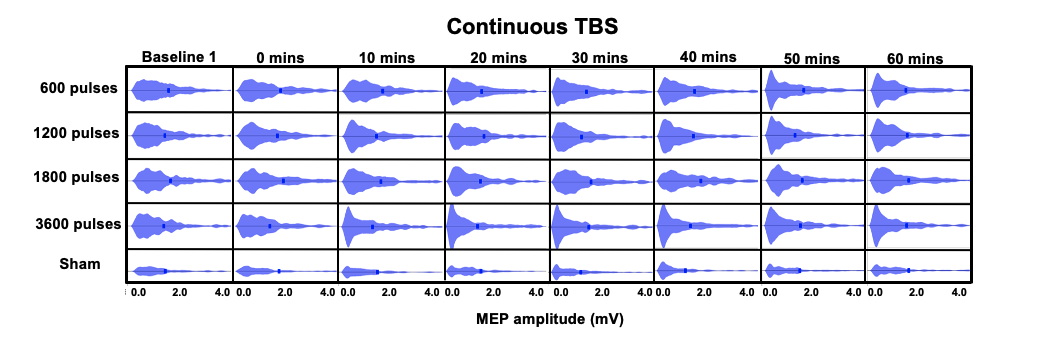


**b)**

*Supplemental Fig. S2: Linear Mixed Effects Model Structure*


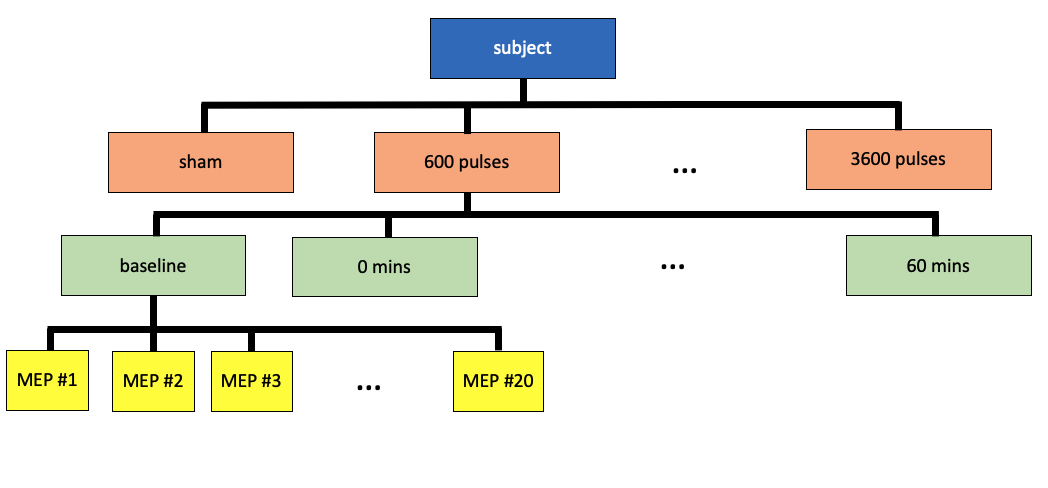


*Supplemental Fig. S3: Linear-mixed effects model prediction of TBS response accounting for resting motor threshold and random effects*


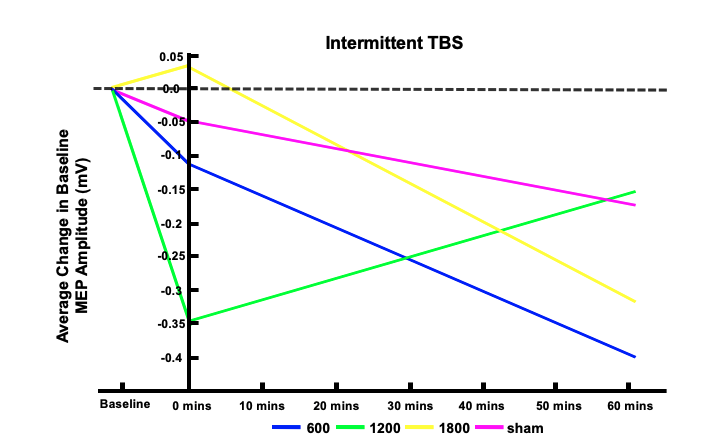


**a)**


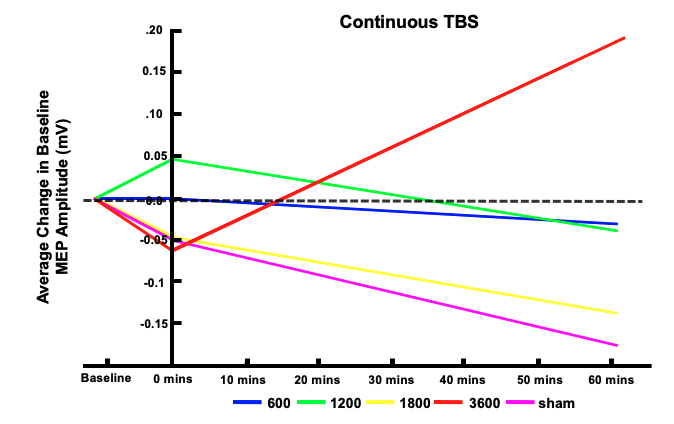


**b)**

*Supplemental Fig. S4: Average change from baseline MEP amplitude over time without*

*outlier correction*
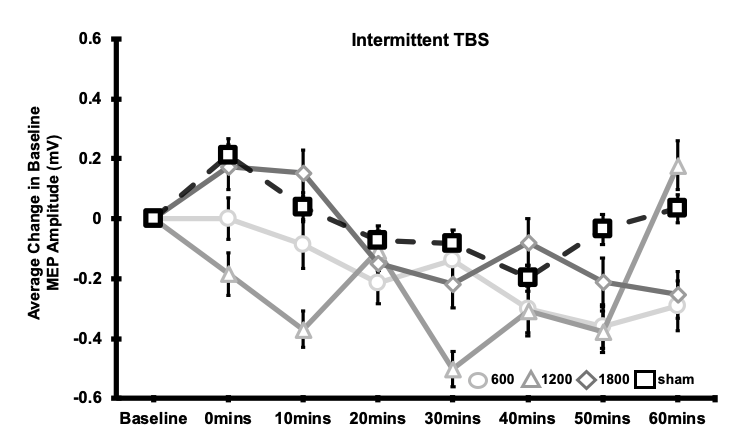

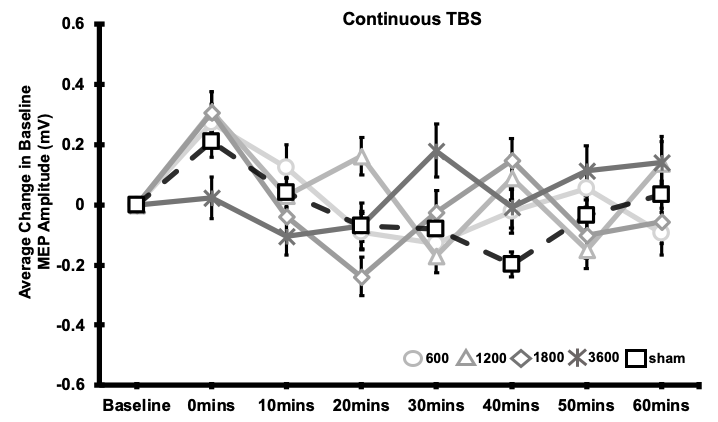


**b)**

**a)**

*Supplemental Fig.* *S5: Individual RMT reliability across visits*

**a)**


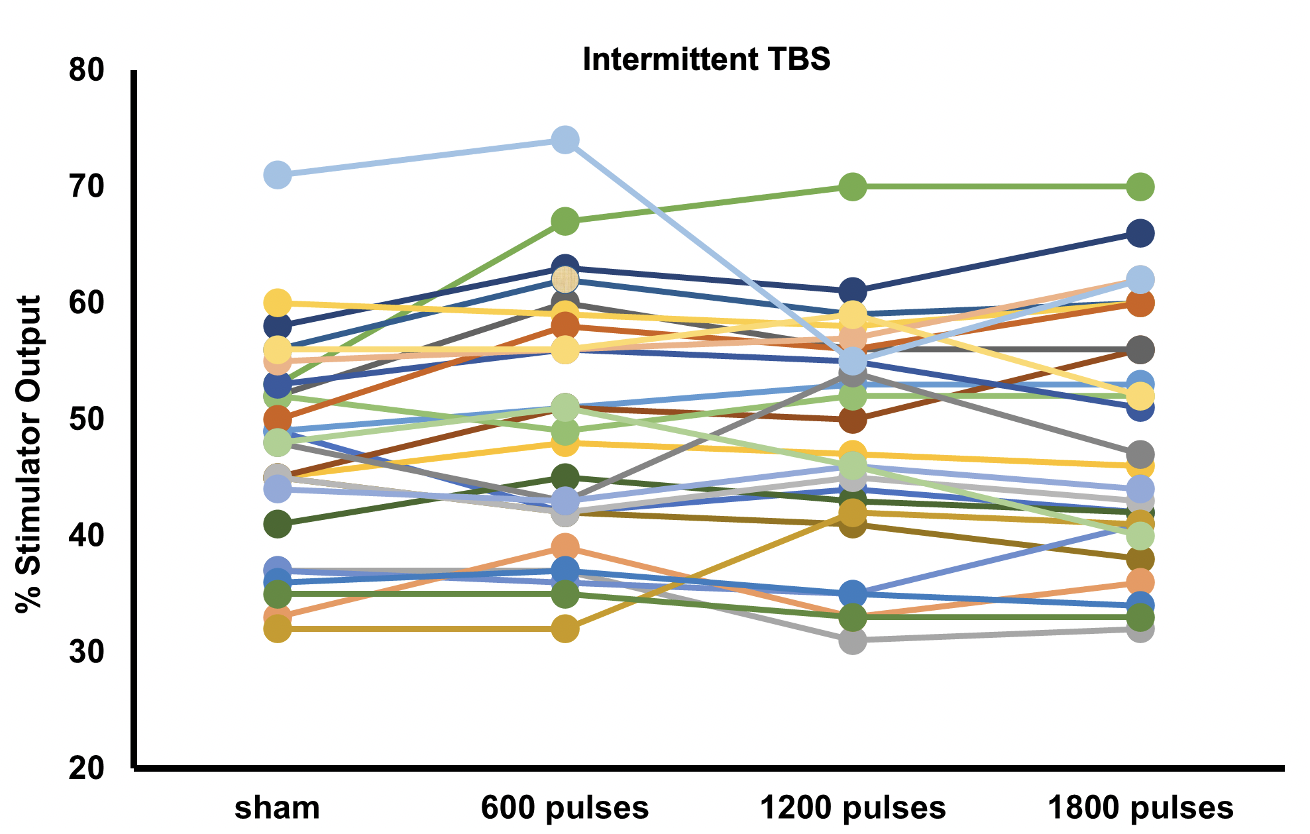


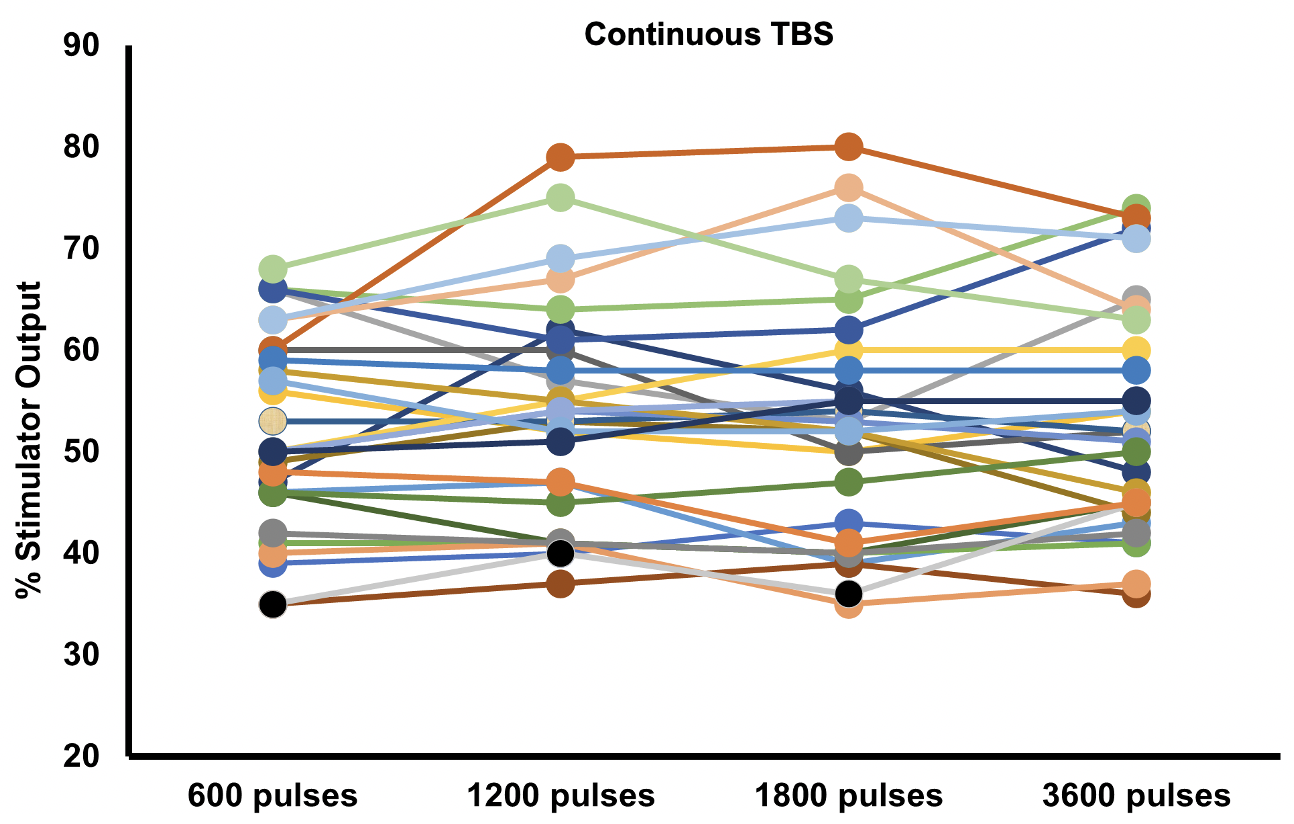


**b)**

*Supplemental Fig.* *S6: Individual AMT reliability across visits*


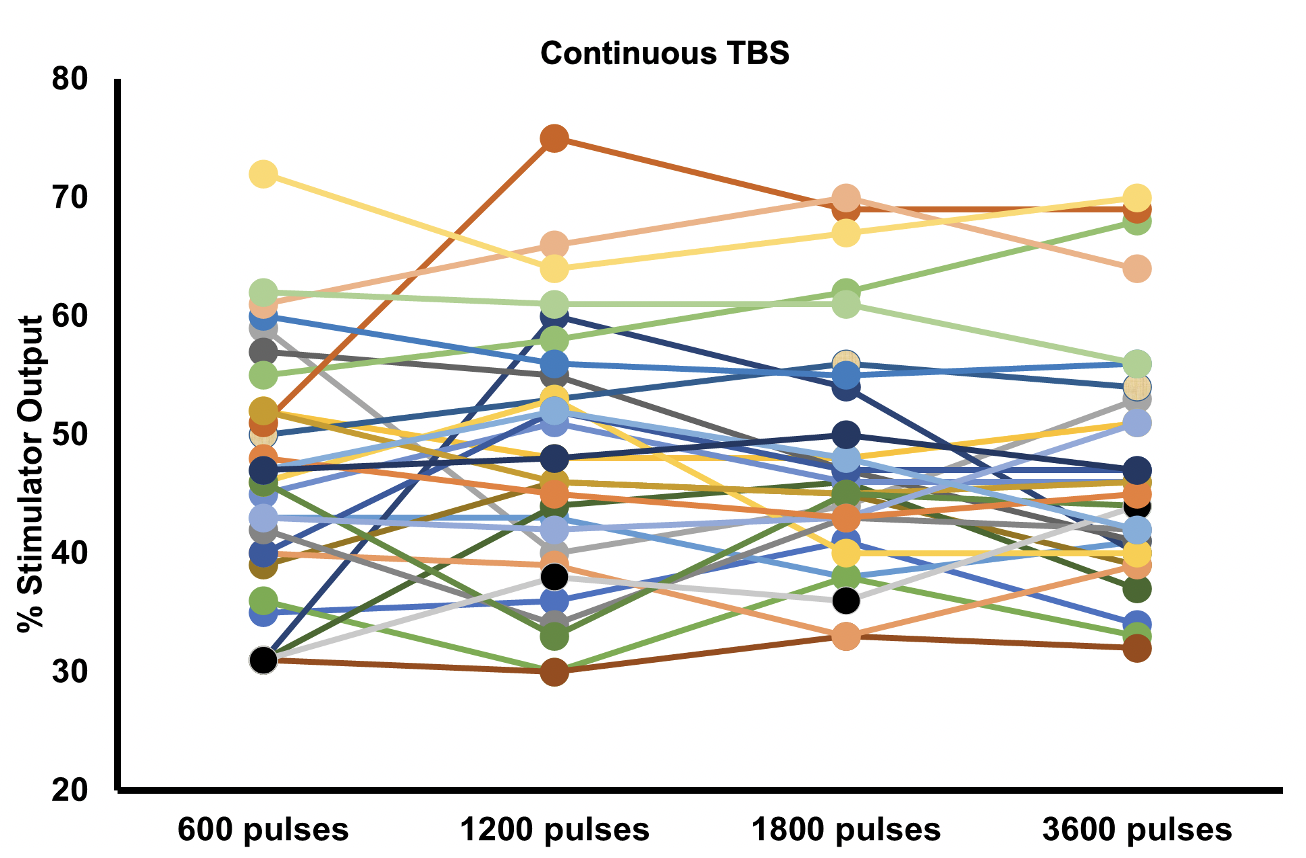

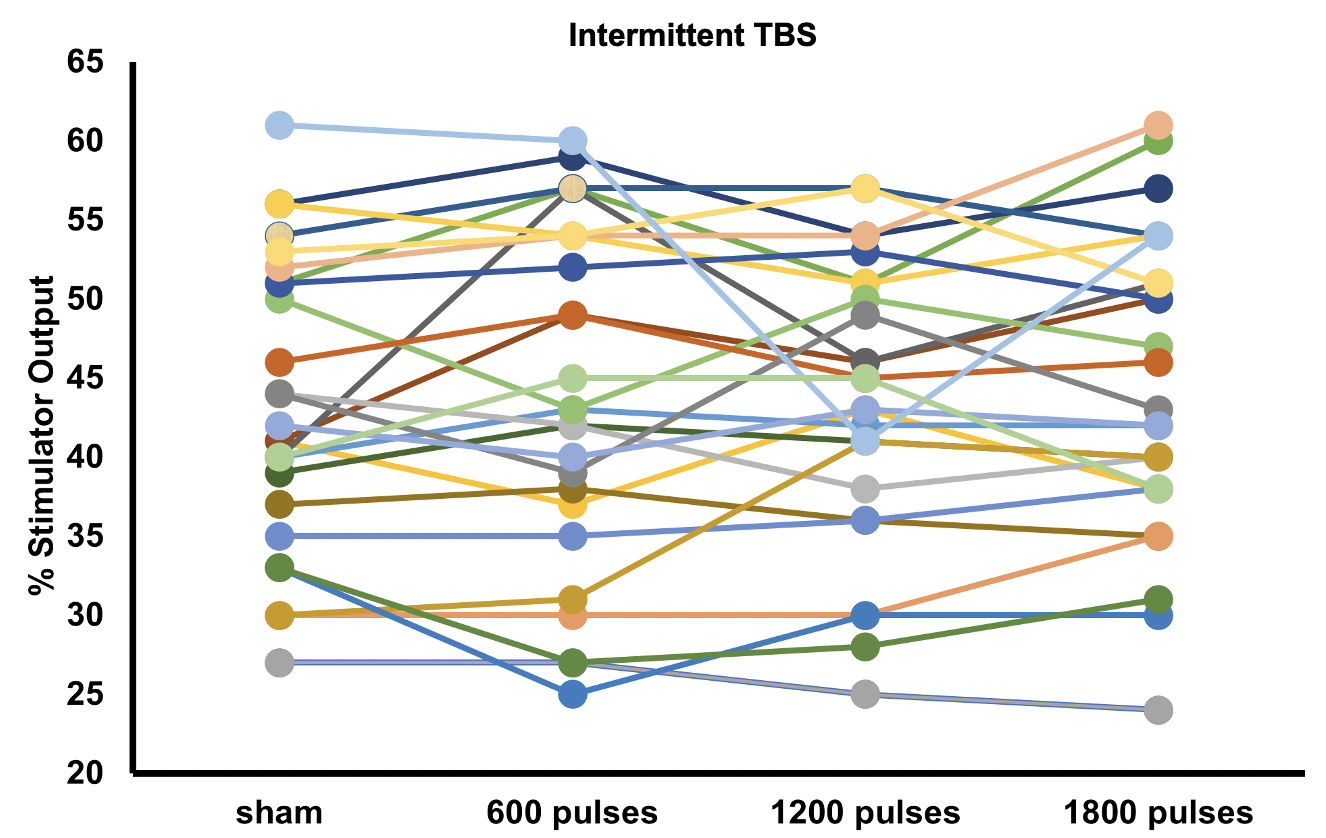


**a)**

**b)**

*Supplemental Fig.* *S7: Individual SI_1mV_ reliability across visits*

**a)**


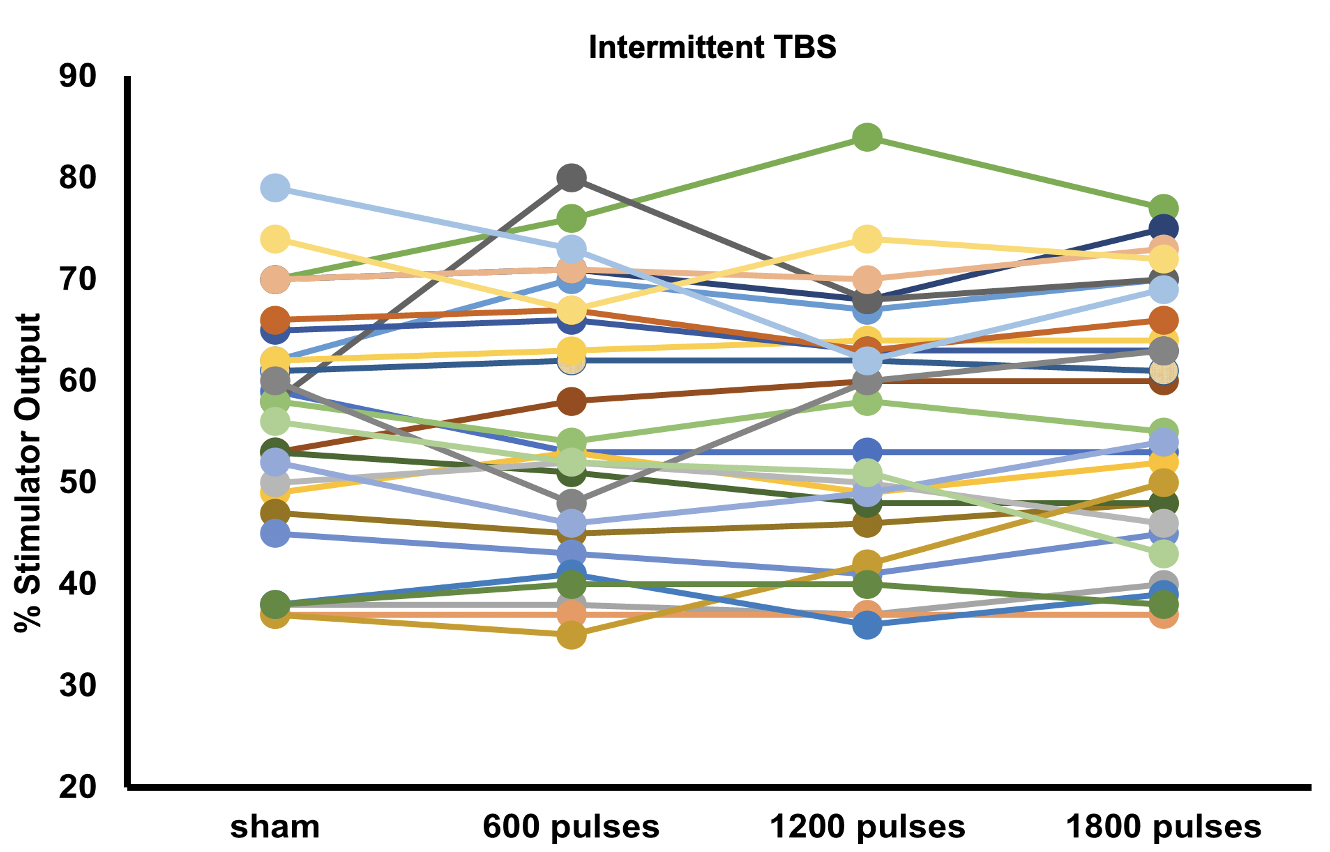


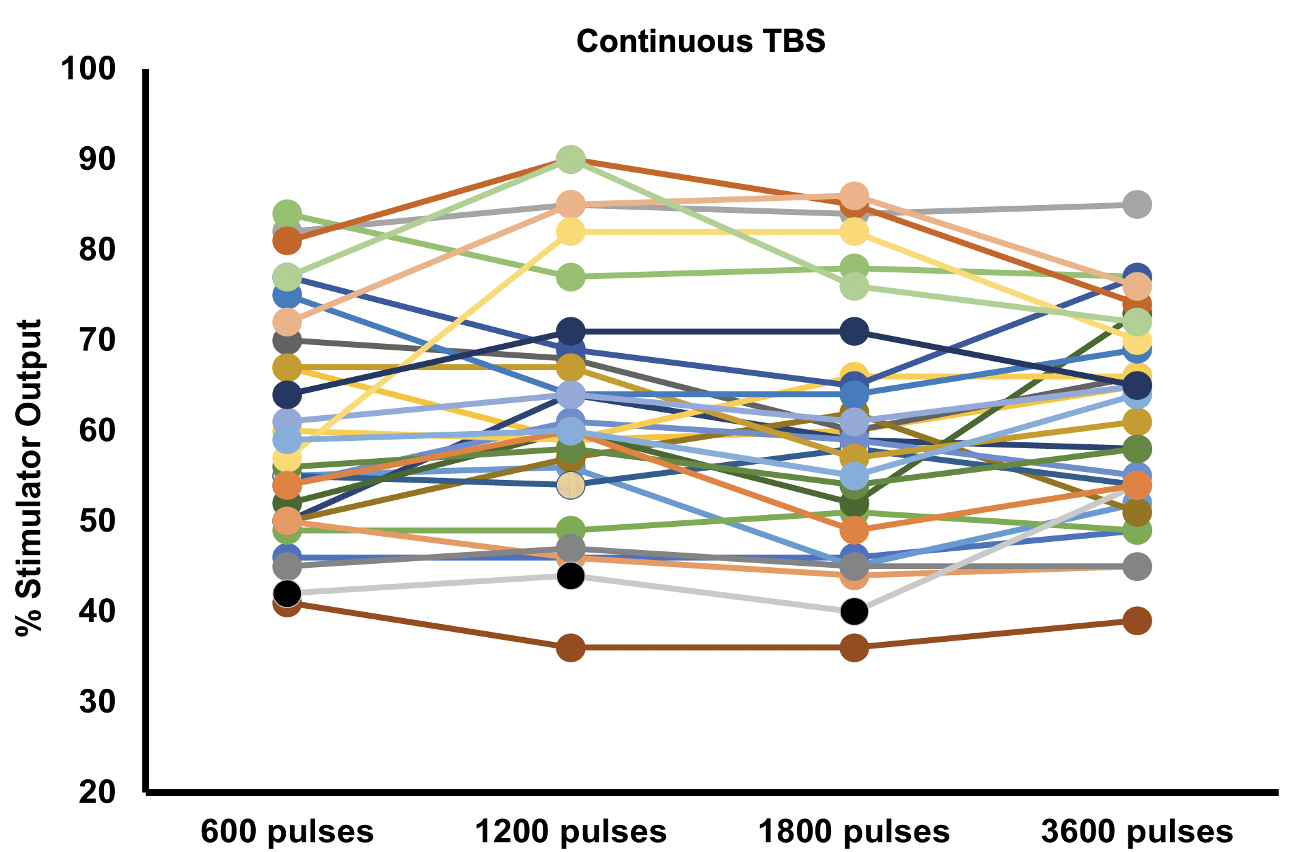


**b)**

*Supplemental Fig.* *S8: Individual Average Baseline MEP amplitude reliability across visits*


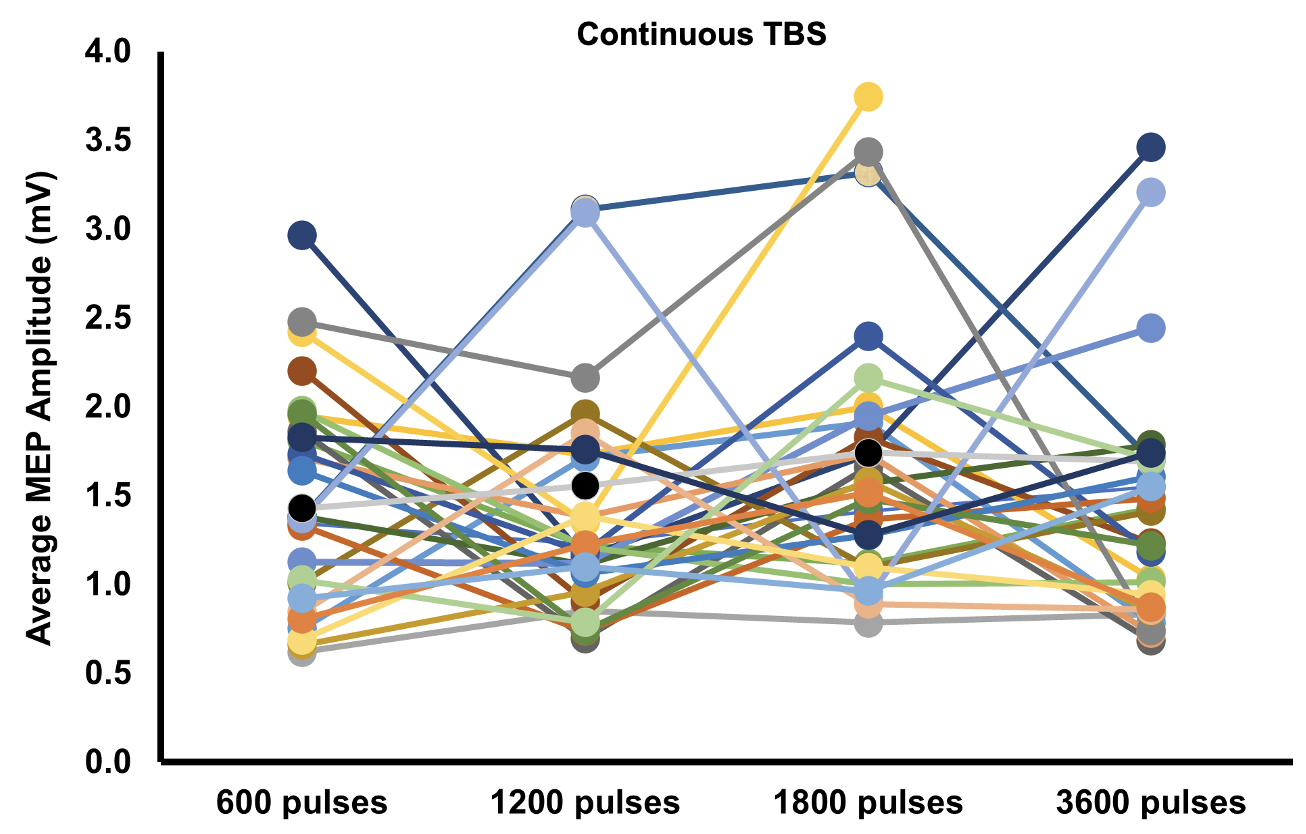

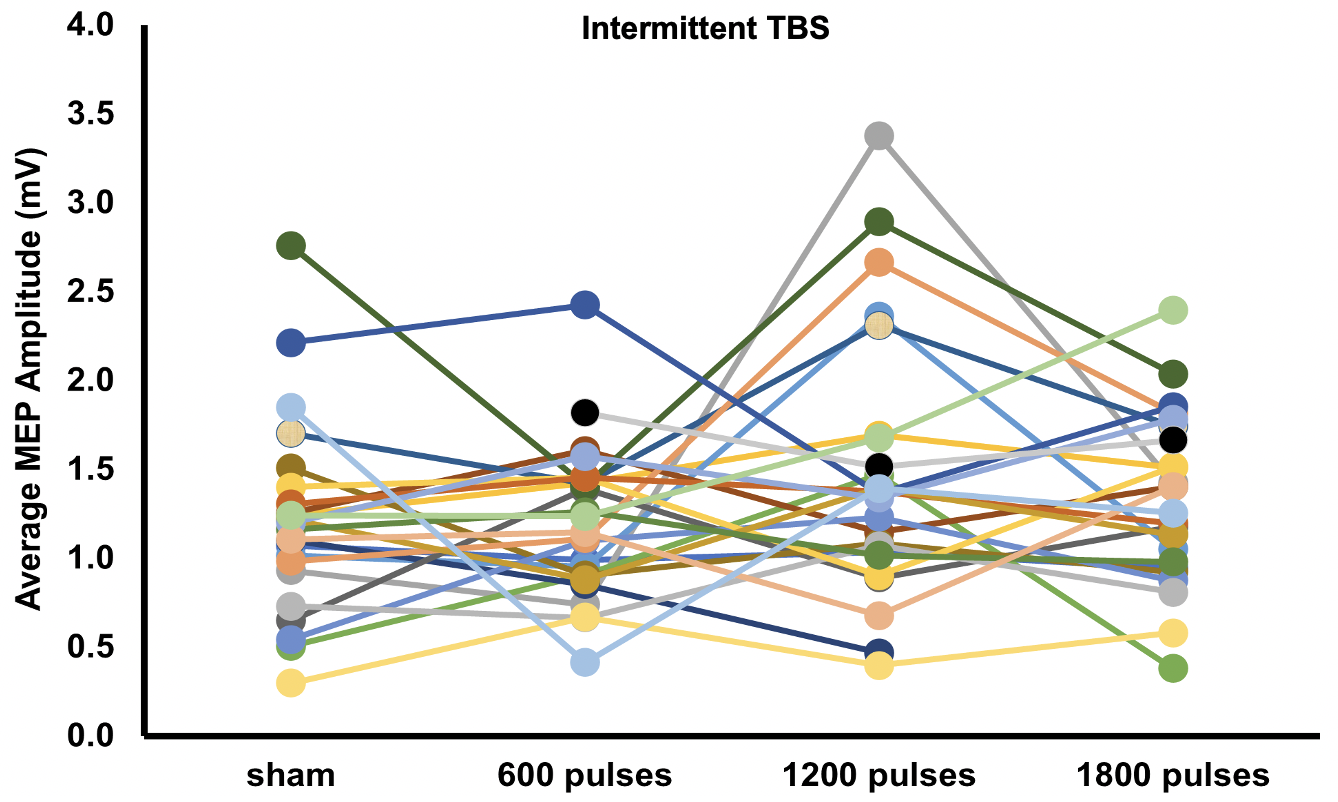


**a)**

**b)**

*Supplemental Table* *S1: Quantification of MEPs included in statistical analysis across treatments*

*Low outlier: MEP amplitudes < 0.3 mV

*High outlier: Change in MEP amplitude > 2.5 STDEV from individual mean change

*Supplemental Table* *S2: Quantification of MEPs included in statistical analysis across participants*
